# Supplementary material for: Telomere Maintenance Mechanisms in a Cohort of High-Risk Neuroblastoma Tumors and Its Relation to Genomic Variants in the TERT and ATRX Genes
Source: Cancers (Basel). 2023 Dec 7;15(24):5732. doi: 10.3390/cancers15245732 (PMC10741428; doi:10.3390/cancers15245732)
Supplement: Supplementary file 1 [file cancers-15-05732-s001.zip › Supplemental Figure S4.pdf]

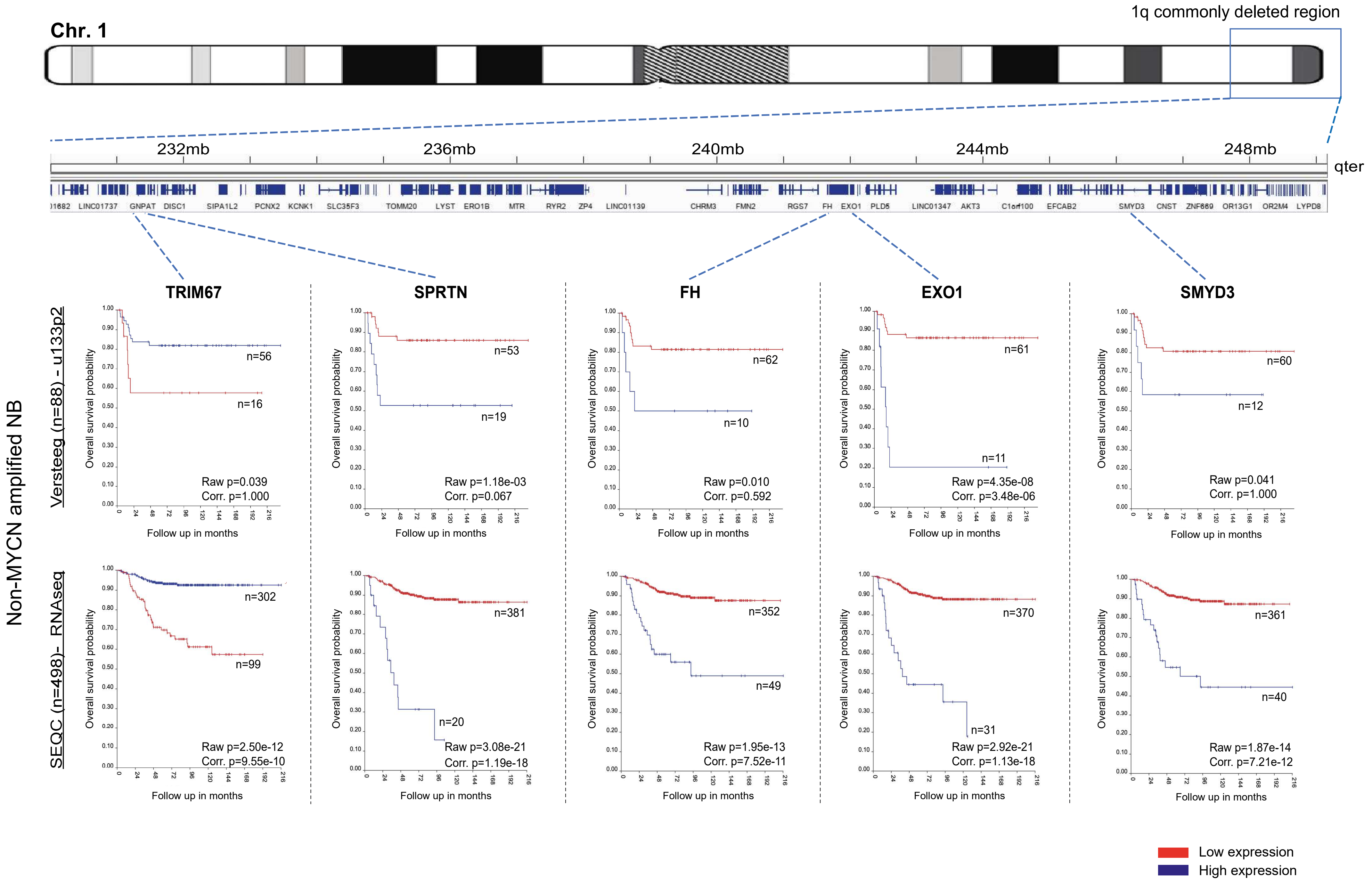

Chr. 1
1q commonly deleted region
232mb
236mb
240mb
244mb
248mb
qter
LINC01737
GNPAT
DISC1
SIPA1L2
PCNX2
KCNK1
SLC35F3
TOMM20
LYST
ERO1B
MTR
RYR2
ZP4
LINC01139
CHRM3
FMN2
RGS7
FH
EXO1
PLD5
LINC01347
AKT3
EFCAB2
SMYD3
CNST
OR13G1
OR2M4
LYPD8
Non-MYCN amplified NB
Versteeg (n=88) - u133p2
SEQC (n=498)- RNAseq
TRIM67
SPRTN
FH
EXO1
SMYD3
Overall survival probability
Follow up in months
n=56
n=16
Raw p=0.039
Corr. p=1.000
n=53
n=19
Raw p=1.18e-03
Corr. p=0.067
n=62
n=10
Raw p=0.010
Corr. p=0.592
n=61
n=11
Raw p=4.35e-08
Corr. p=3.48e-06
n=60
n=12
Raw p=0.041
Corr. p=1.000
n=302
n=99
Raw p=2.50e-12
Corr. p=9.55e-10
n=381
n=20
Raw p=3.08e-21
Corr. p=1.19e-18
n=352
n=49
Raw p=1.95e-13
Corr. p=7.52e-11
n=370
n=31
Raw p=2.92e-21
Corr. p=1.13e-18
n=361
n=40
Raw p=1.87e-14
Corr. p=7.21e-12
Low expression
High expression
