# Supplementary figures and images for: Telomere Maintenance Mechanisms in a Cohort of High-Risk Neuroblastoma Tumors and Its Relation to Genomic Variants in the TERT and ATRX Genes
Source: Cancers (Basel). 2023 Dec 7;15(24):5732. doi: 10.3390/cancers15245732 (PMC10741428; doi:10.3390/cancers15245732)

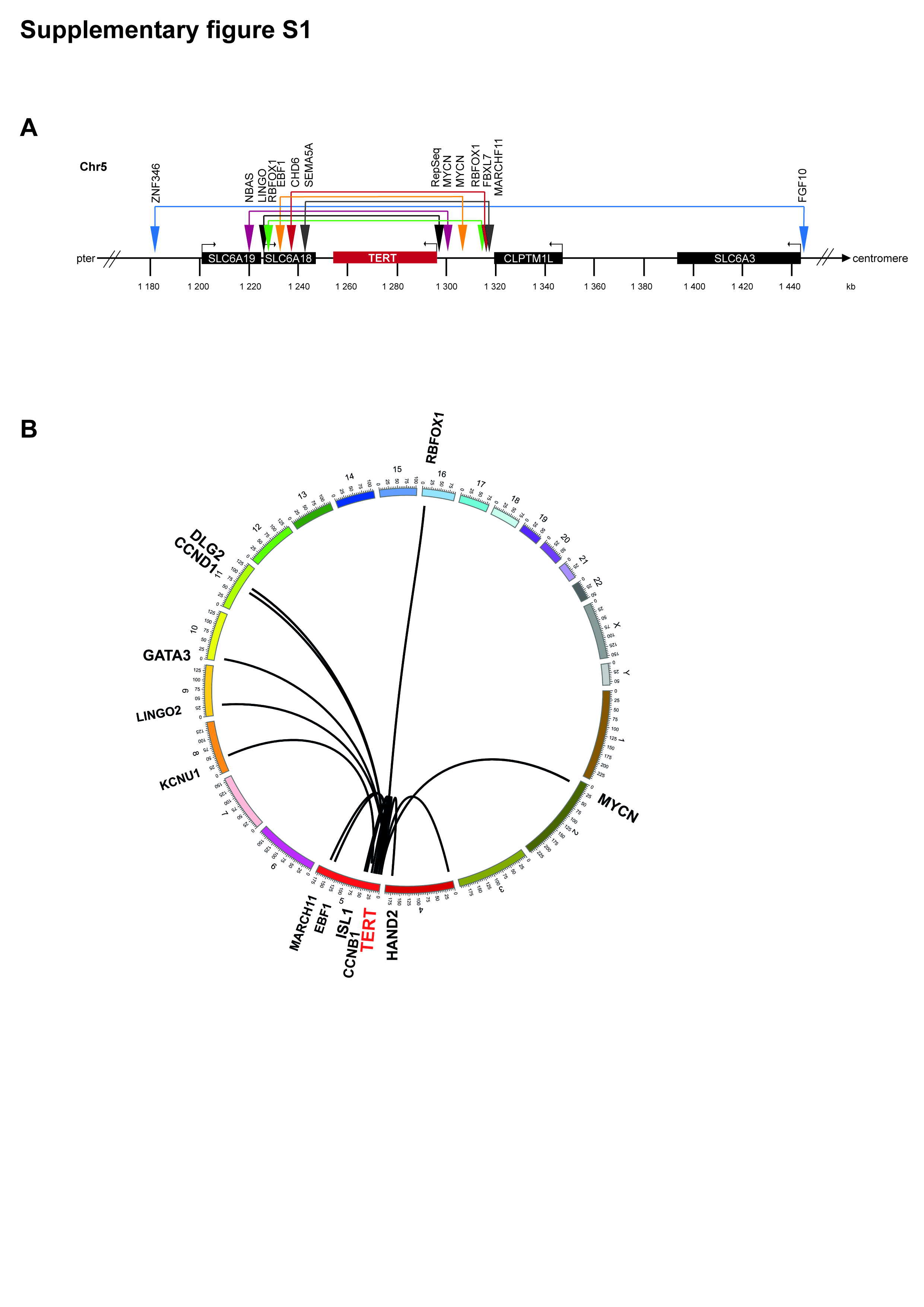

Supplement: Supplementary file 1 [file cancers-15-05732-s001.zip › Supplemental figure S1.tif]

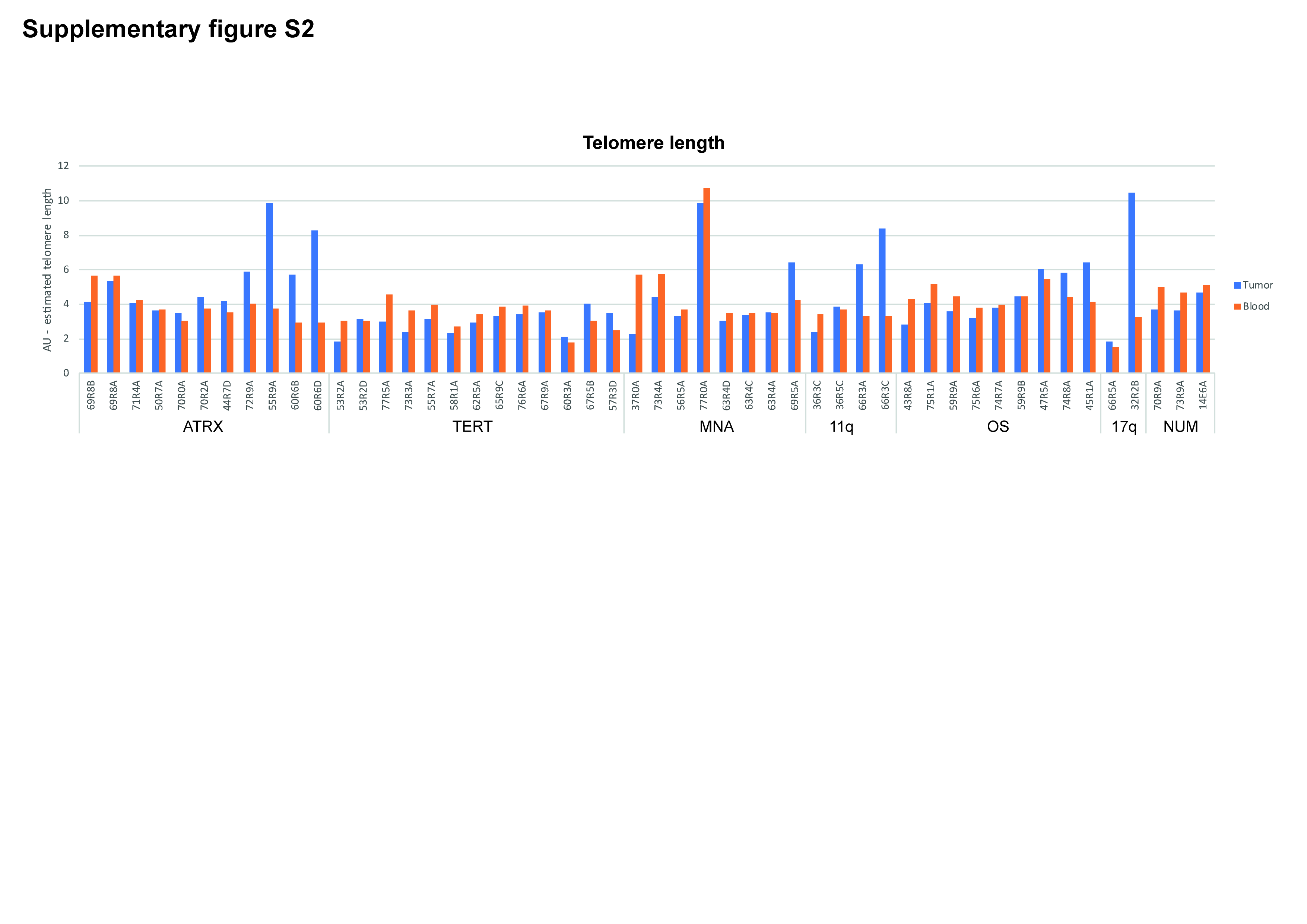

Supplement: Supplementary file 1 [file cancers-15-05732-s001.zip › Supplemental figure S2.tif]

Supplemnetary figure 3. Uncropped c-circle assay blots for samples included in figure 3.

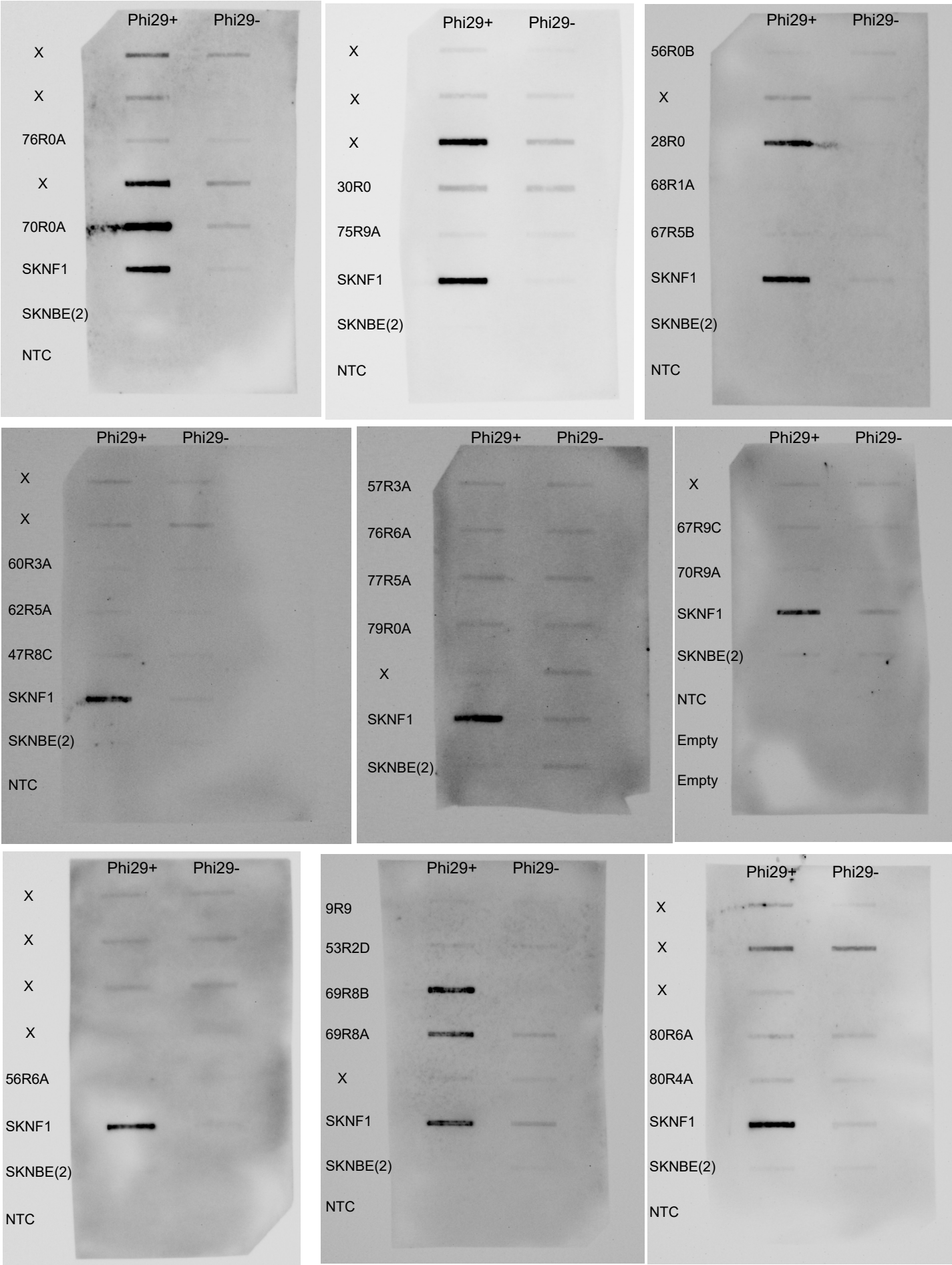

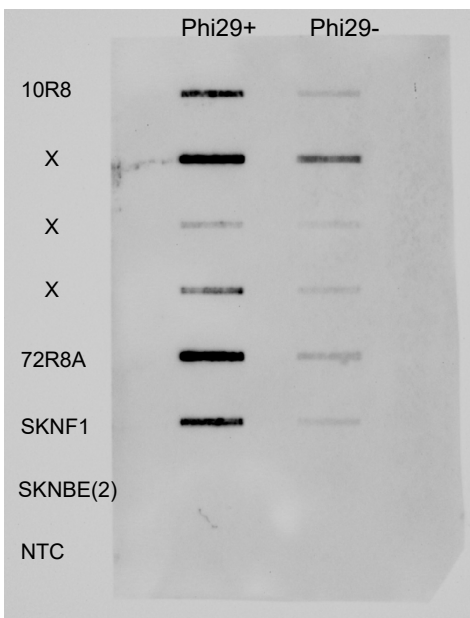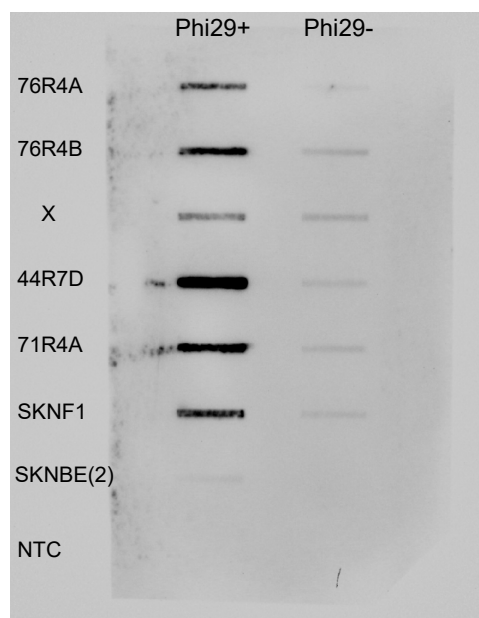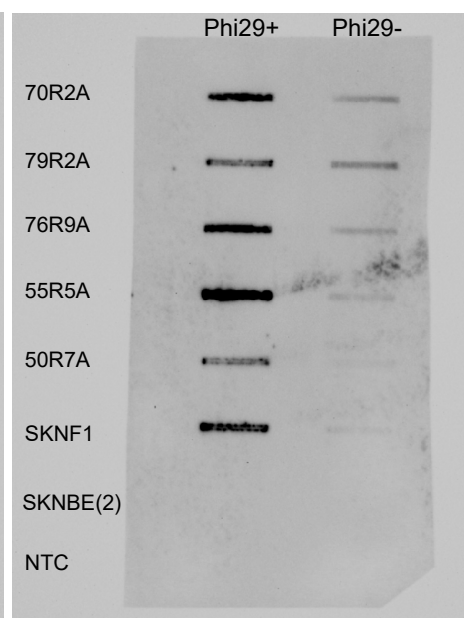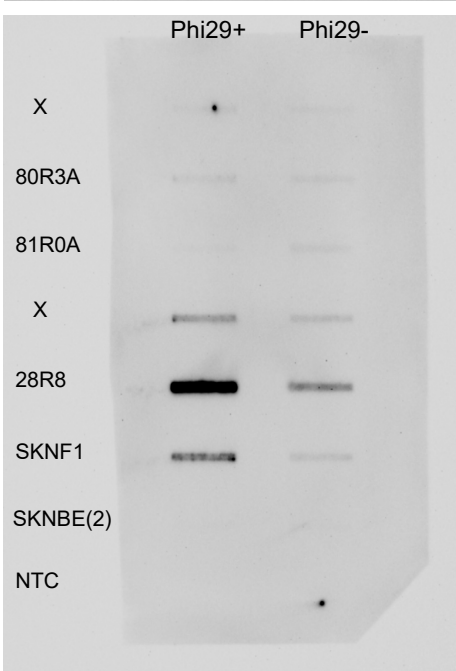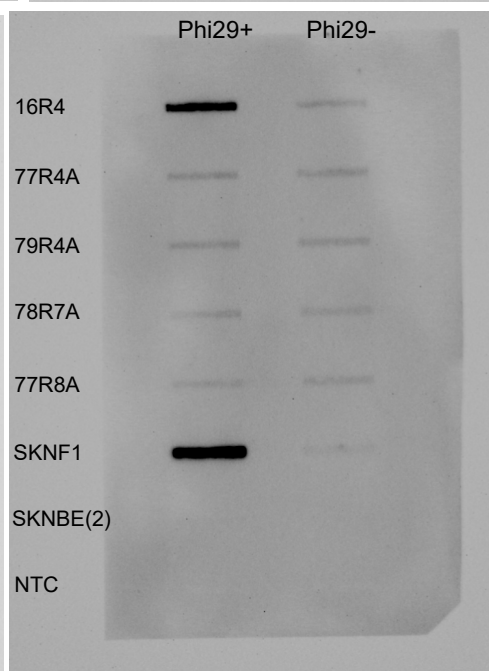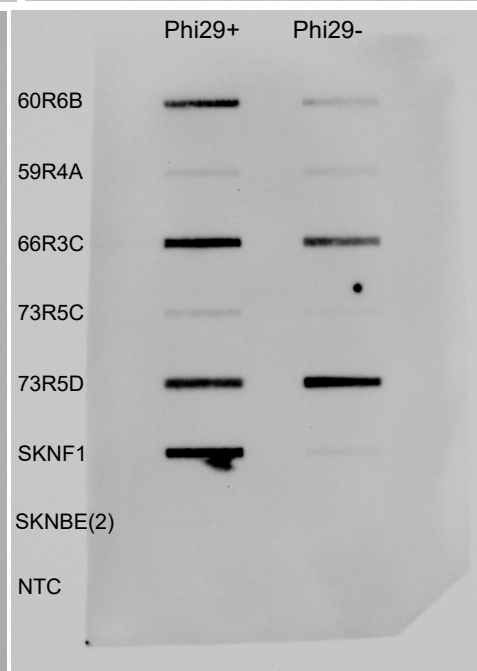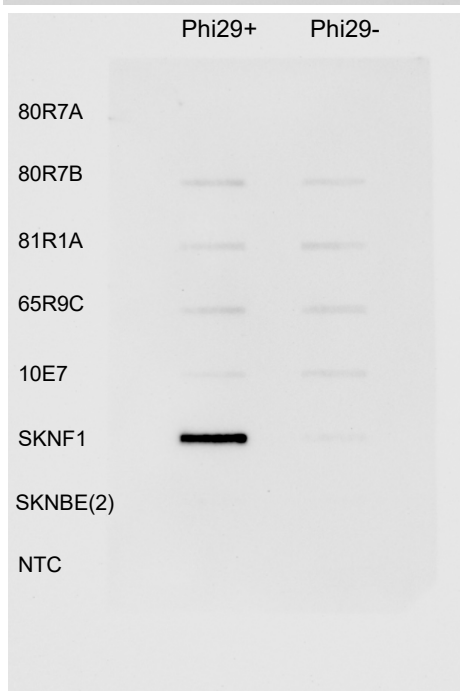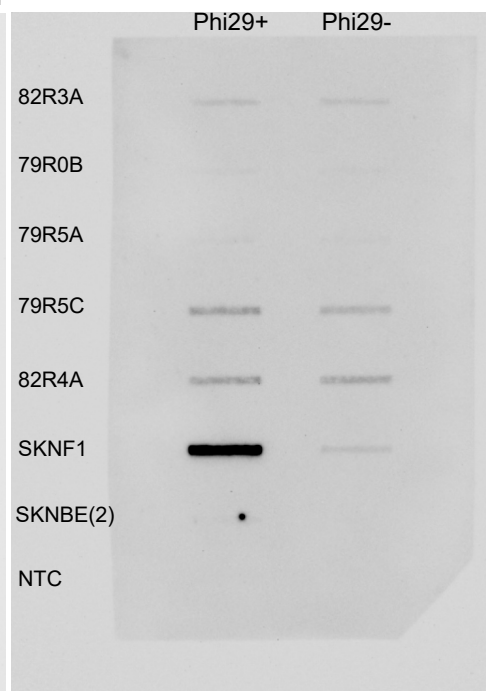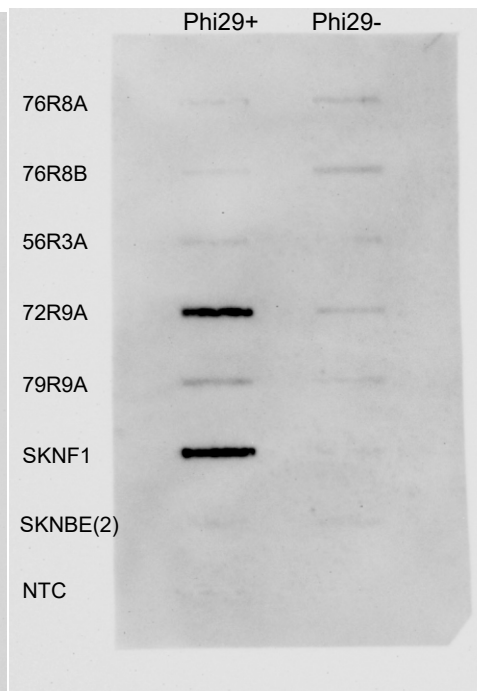

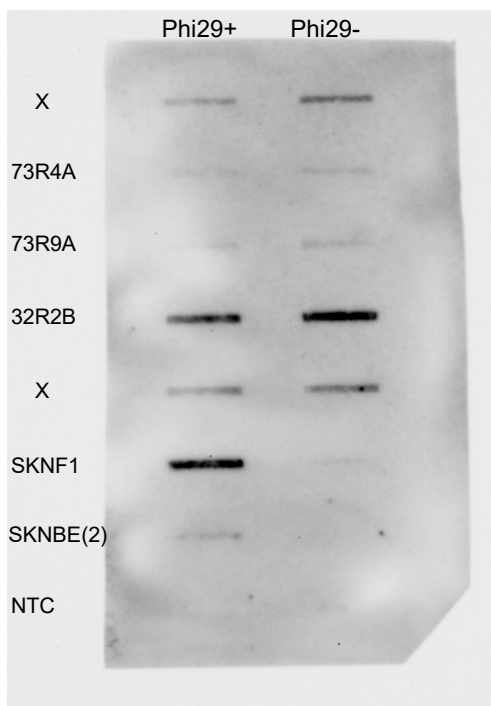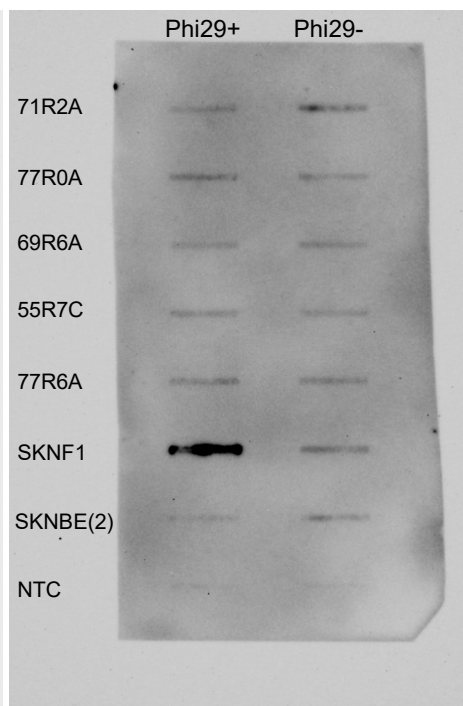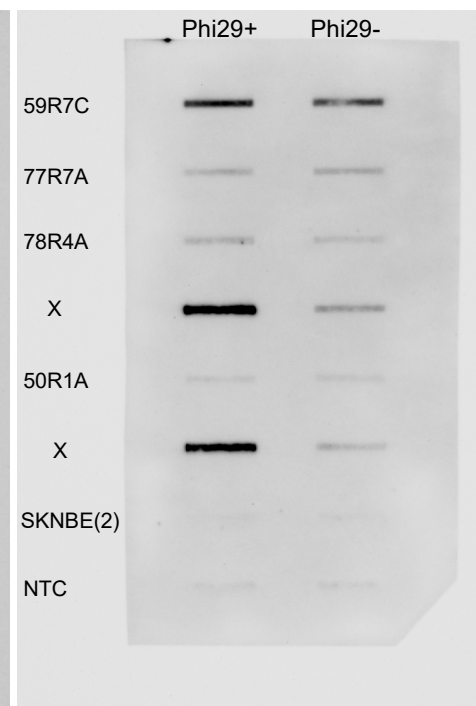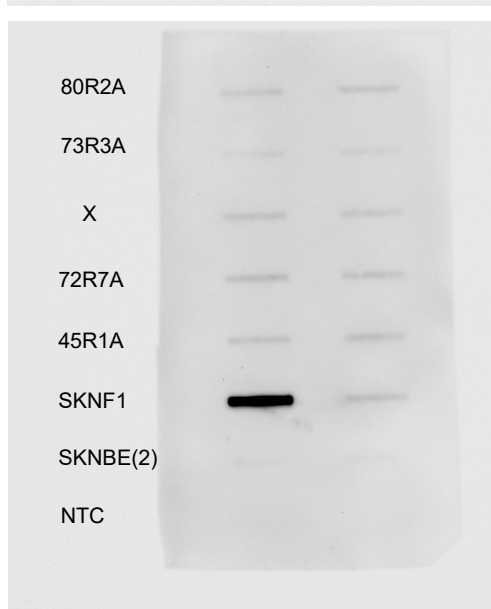

Supplement: Supplementary file 1 [file cancers-15-05732-s001.zip › Supplemental Figure S3.pdf]
